# Revealing shared molecular markers and mechanisms in colorectal cancer and COVID-19 through bioinformatics and machine learning

**DOI:** 10.1093/bib/bbag065

**Published:** 2026-02-23

**Authors:** Hairui Wang, Haodong Yao, Wenchao Niu, Rongchang Xing, Lu Cai, Zuoxin Xi, Shichen Gao, Lina Zhao

**Affiliations:** School of Science, China University of Geosciences, Xueyuan Road 29, Haidian District, Beijing 100083, China; Multi-disciplinary Research Division, Institute of High Energy Physics Chinese Academy of Sciences, 19B Yuquan Road, Shijingshan District, Beijing 100049, China; Multi-disciplinary Research Division, Institute of High Energy Physics Chinese Academy of Sciences, 19B Yuquan Road, Shijingshan District, Beijing 100049, China; University of Chinese Academy of Sciences, 19A Yuquan Road, Shijingshan District, Beijing 100049, China; Multi-disciplinary Research Division, Institute of High Energy Physics Chinese Academy of Sciences, 19B Yuquan Road, Shijingshan District, Beijing 100049, China; School of Science, China University of Geosciences, Xueyuan Road 29, Haidian District, Beijing 100083, China; Multi-disciplinary Research Division, Institute of High Energy Physics Chinese Academy of Sciences, 19B Yuquan Road, Shijingshan District, Beijing 100049, China; School of Science, China University of Geosciences, Xueyuan Road 29, Haidian District, Beijing 100083, China; Multi-disciplinary Research Division, Institute of High Energy Physics Chinese Academy of Sciences, 19B Yuquan Road, Shijingshan District, Beijing 100049, China; Multi-disciplinary Research Division, Institute of High Energy Physics Chinese Academy of Sciences, 19B Yuquan Road, Shijingshan District, Beijing 100049, China; School of Information Engineering, Minzu University of China, 27 Zhongguancun South Avenue, Beijing 100083, China; School of Science, China University of Geosciences, Xueyuan Road 29, Haidian District, Beijing 100083, China; Multi-disciplinary Research Division, Institute of High Energy Physics Chinese Academy of Sciences, 19B Yuquan Road, Shijingshan District, Beijing 100049, China; University of Chinese Academy of Sciences, 19A Yuquan Road, Shijingshan District, Beijing 100049, China

**Keywords:** comorbidity, bioinformatics, machine learning, COVID-19, colorectal cancer

## Abstract

Colorectal cancer (CRC) and Coronavirus Disease 2019 (COVID-19) are distinct diseases that may share overlapping molecular mechanisms, particularly in immune dysregulation. However, the specific regulatory pathways driving this shared pathophysiology have remained elusive, as prior studies have been limited by single-level data. To dissect this common pathobiology, we implemented a synergistic computational framework, integrating bulk transcriptomics with single-cell data. Through a multi-tiered analysis pipeline employing differential expression, weighted gene co-expression networks, and machine learning–based feature selection, we pinpointed a core molecular signature of 31 shared hub genes. Among these, four core candidates—GPR15, PTGDR2, FCER1A, and MAL—were significantly downregulated, a finding robustly associated with impaired CD8^+^ T cell infiltration. Delving deeper into the regulatory architecture using a modified weighted out-degree centrality algorithm, we constructed an integrated transcription factor–microRNA–target network. Network analysis revealed upregulation of p53 and downregulation of miR-3619-5p as possible drivers of immune dysfunction. Finally, E-4031 was identified through molecular simulation as a potential therapeutic agent targeting all four core genes. These findings uncover a shared regulatory axis involving immune suppression and transcriptional disruption, and provide promising diagnostic and therapeutic targets for CRC and COVID-19.

## Graphical Abstract

**Graphical Abstract:**
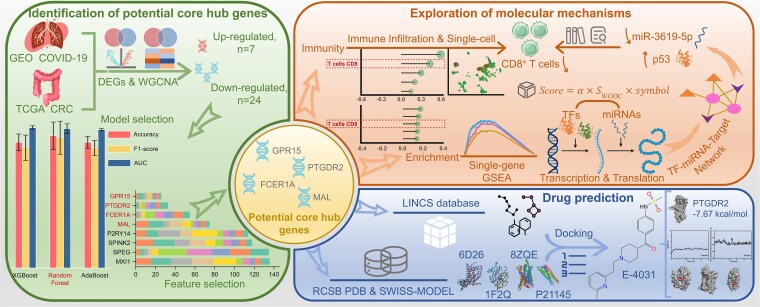

## Introduction

Colorectal cancer (CRC) stands as a formidable global health challenge. In 2022, it accounted for ~9.6% of all cancer cases worldwide, ranking as the third most commonly diagnosed cancer and the second leading cause of cancer-related deaths [1]. In China, CRC presents an equally serious burden, ranking as the third most common malignancy [2], and there were 517 100 new CRC cases in 2022, ranking second in incidence and fourth in mortality among all cancers [3]. Despite significant progress in conventional therapies, clinical outcomes are often hampered by an incomplete understanding of its pathogenesis and a lack of reliable early biomarkers, which complicates the removal of precursor polyps and elevates the risk of recurrence and metastasis [4, 5].

Coronavirus disease 2019 (COVID-19), caused by SARS-CoV-2, introduced another layer of complexity to global health. The pandemic spread rapidly worldwide since late 2019, with 414 179 confirmed cases and 18 440 deaths reported across 197 countries by March 2020 [6]. SARS-CoV-2 enters host cells by binding to the ACE2 receptor on the cell surface [7]. Beyond the respiratory system, COVID-19 affects the digestive and nervous systems [8]; severe cases can present with extensive mucosal sloughing in the small intestine and colon [9], severe colonic ischemia [10], and colonic perforation [11]. A critical molecular link has been established: ACE2 is upregulated in CRC and is associated with immune infiltration in colorectal adenocarcinoma [12, 13]. Cancer patients, including those with CRC, have higher infection risk and poorer outcomes [14], and CRC patients are often at increased risk of SARS-CoV-2 infection [12]. SARS-CoV-2 infection can disrupt gut microbiota [15], potentially increasing CRC risk via dysbiosis [16]. These findings indicate substantial interplay between COVID-19 and CRC, warranting further investigation into shared molecular mechanisms.

In light of these clinical observations, transcriptomic studies have begun to probe the shared pathobiology of CRC and COVID-19. For instance, pan-cancer analyses have confirmed the upregulation of ACE2 in CRC tissues and its correlation with immune cell infiltration [17]. Chen *et al.*, using intestinal organoid infection datasets, has identified differentially expressed genes that reveal intersections between viral infection and tumor-related pathways [18]. However, these foundational studies have been limited by their broad scope or their struggle to extract clear signals from the inherent noise of high-dimensional omics data. As a result, a comprehensive and focused investigation into the shared molecular signatures, comorbidity mechanisms, and therapeutic targets of CRC and COVID-19 is still lacking. Consequently, identifying the key genes and regulatory networks that define this comorbidity remains a significant challenge.

To address this, our study was designed to systematically dissect the shared diagnostic genes, molecular interactions, and potential therapeutic avenues between CRC and COVID-19. We implemented a multi-stage integrative analytical framework to identify common transcriptomic signatures, prioritize clinically relevant biomarkers, and computationally screen for therapeutic candidates. The analytical workflow consists of three main modules. We first employed differential expression analysis and Weighted Gene Co-expression Network Analysis (WGCNA) to identify disease-relevant gene modules. This was followed by the application of robust machine learning algorithms to distill a high-confidence set of core hub genes. These genes were further validated through spatial localization and expression consistency in independent datasets and single-cell transcriptomic profiles. We then investigated the biological roles of these core genes through single-cell analysis, immune infiltration analysis, functional enrichment, and upstream regulatory analysis. A key objective was the construction of a comprehensive regulatory network to elucidate how these genes orchestrate immune signaling within the context of CRC and COVID-19 comorbidity. Finally, we performed a computational screen for candidate drug compounds. The interactions between promising agents and the core gene targets were evaluated using molecular docking and molecular dynamics simulations to verify their therapeutic potential from a mechanistic standpoint. Collectively, this study not only reveals the molecular convergence between CRC and COVID-19 but also provides a high-confidence resource for biomarker discovery and drug repositioning. The findings underscore the importance of cross-disease multi-omics integration in deciphering complex disease networks and advancing translational research.

## Materials and Methods

### Overview of methodological framework

**Figure 1:**
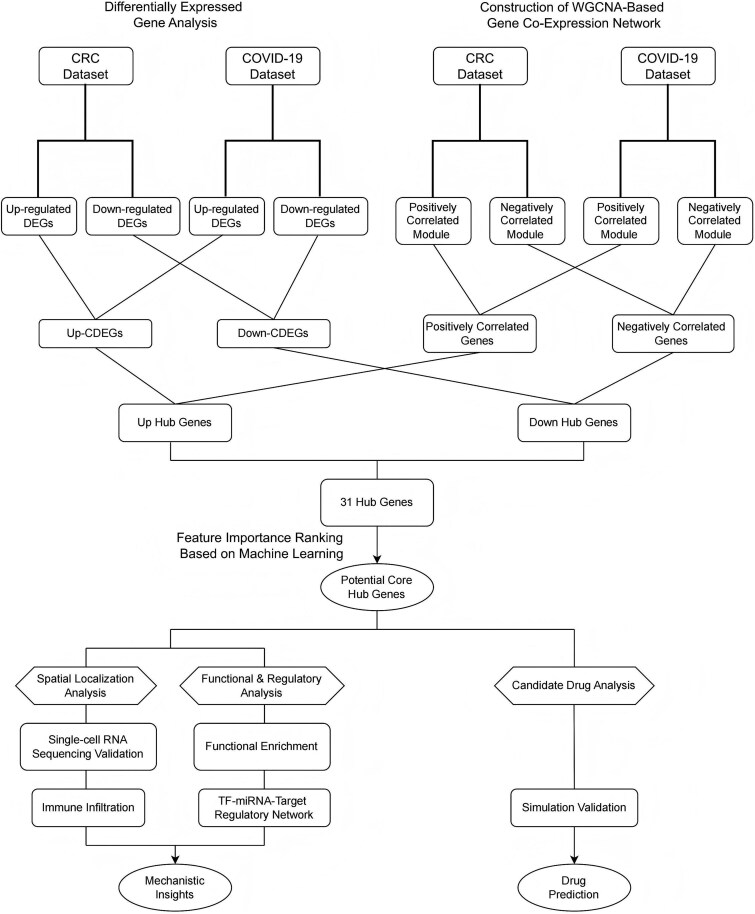
Overview of the analytical framework. The workflow integrates transcriptomic analysis, core gene identification, molecular mechanism exploration, and drug prediction to investigate shared molecular markers and mechanisms between COVID-19 and CRC.

The overall workflow of this study is illustrated in Fig. 1 and consists of three interconnected modules designed to enhance analytical robustness and biological interpretability. First, bulk RNA-seq data from Gene Expression Omnibus (GEO) and The Cancer Genome Atlas (TCGA) were analyzed using differential expression analysis and WGCNA, providing complementary strategies to identify genes with both statistical significance and network relevance. The intersected genes were then subjected to machine learning–based model selection and feature prioritization to identify potential core hub genes. In the mechanistic exploration layer, we investigated functional roles through single-cell spatial localization, immune infiltration analysis, single-gene gene set enrichment analysis (GSEA), and construction of a transcription factor–microRNA–target (TF–miRNA–Target) regulatory network. Finally, candidate therapeutics were predicted using the Library of Integrated Network-based Cellular Signatures (LINCS) database and validated via molecular docking and molecular dynamics simulations. This multi-layered integrative strategy bridges transcriptomic discovery with mechanistic insight and therapeutic prediction, offering a systematic framework for identifying key genes in CRC–COVID-19 comorbidity.

### Data collection

Three publicly available datasets were utilized in this study.

- COVID-19 transcriptome: the high-throughput RNA-seq dataset for COVID-19 (GSE152641) was obtained from the National Center for Biotechnology Information (NCBI) GEO database [19]. This dataset was selected for its relatively large sample size, well-annotated clinical metadata, and its frequent usage in recent COVID-19 transcriptomic studies.
- Colorectal cancer transcriptome: the CRC dataset was retrieved from the University of California, Santa Cruz (UCSC) Xena platform [20], which integrates standardized TCGA pan-cancer data. Specifically, we utilized the TCGA-COAD RNA-seq dataset, chosen for its high sequencing depth, large cohort size, and comprehensive clinical annotation.
- Single-cell validation dataset: the single-cell RNA-seq dataset GSE146771 was obtained from the GEO database. This dataset was selected for its suitability in validating the cell-type-specific expression patterns of candidate genes identified from the bulk RNA-seq analysis.

A detailed summary of the datasets, including accession numbers, sequencing platforms, sample composition, and references, is provided in Table S1.

### Identification and enrichment analysis of common differentially expressed genes (DEGs) between COVID-19 and colorectal cancer

To identify transcriptional alterations between disease and control groups, we performed differential expression analysis. This approach fits a linear model for each gene and employs an empirical Bayes method to improve the robustness of variance estimation, which is particularly advantageous for small-sample datasets. The basic model is formulated as follows:


(1) \begin{equation*} {Y}_{gi}={\mu}_g+{\beta}_g{X}_i+{\varepsilon}_{gi} \end{equation*}

where ${Y}_{gi}$ is the expression of gene *g* in sample *i*, ${\beta}_g$ represents the condition effect, and ${\varepsilon}_{gi}$ is the residual error. Detailed parameter settings are provided in Supplementary Note S1.

To enhance robustness, DEGs were identified independently in both COVID-19 and CRC datasets, followed by intersection analysis to obtain common DEGs (CDEGs). This approach highlights shared transcriptional perturbations, which may reflect convergent pathophysiological mechanisms.

### Construction of Weighted Gene Co-expression Network Analysis for co-expression network

To move beyond individual gene changes and identify functionally related gene communities, we employed WGCNA. This method [21] constructs a scale-free gene co-expression network by first calculating pairwise Pearson correlations between all gene expression profiles. These correlations are then transformed into an adjacency matrix ${a}_{ij}$,using a soft-thresholding power (*b*), which amplifies strong correlations while penalizing weak ones, as shown in Equation (2):


(2) \begin{equation*} {\displaystyle \begin{array}{c}{a}_{ij}={\left| cor\left({x}_i,{x}_j\right)\right|}^{\beta}\end{array}} \end{equation*}

The adjacency matrix is subsequently converted into a Topological Overlap Matrix (TOM), which provides a more robust measure of network interconnectedness by considering both direct and shared neighbors, as shown in Equation (3):


(3) \begin{equation*} {\displaystyle \begin{array}{c}{TOM}_{ij}=\frac{l_{ij}+{a}_{ij}}{\min \left({k}_i,{k}_j\right)+1-{a}_{ij}}\end{array}} \end{equation*}

where ${l}_{ij}=\sum_u{a}_{iu}{a}_{uj}$ represents the number of shared neighbors of genes *i* and gene *j*, and ${k}_i=\sum_u{a}_{iu}$ is the connectivity of gene *i*.

Hierarchical clustering based on TOM dissimilarity was then used to group genes into distinct co-expression modules. The association of each module with disease status was quantified by correlating the module eigengene (the first principal component) with clinical traits. This network-based approach complements differential expression analysis; by intersecting DEGs with disease-associated WGCNA modules, we prioritized genes that are not only statistically significant but also topologically central within biologically coherent networks, thereby enhancing the selection of high-confidence hub genes.

### Identification of potential core hub genes through machine learning

To further refine the selection of key genes beyond statistical significance and network topology, we employed machine learning as the final layer of a multi-stage screening pipeline. While differential expression analysis identifies expression-level changes and WGCNA captures coregulation patterns, machine learning offers a complementary advantage by modeling complex, nonlinear relationships in high-dimensional data. Specifically, the expression profiles of candidate genes were used—derived from the intersection of DEG and WGCNA analyses—as input features, with disease status (disease versus control) as the output label. Details are provided in Supplementary Note S2. Several supervised learning algorithms, including XGBoost, Random Forest, AdaBoost, LightGBM, Bagging, and Decision Tree, were evaluated and compared to determine the optimal model for this classification task. These methods were chosen for their demonstrated effectiveness in modeling high-dimensional omics data and capturing complex feature interactions. By integrating statistical significance, network topology, and model-based feature relevance, this approach allowed for a more reliable identification of potential core hub genes, enhancing both the interpretability and biological relevance of the findings.

To evaluate feature importance, five independent metrics were used for each dataset, including the chi-square test [22], recursive feature elimination [23], *F*-test [24], SHAP [25], and random forest feature importance [26], generating five sets of gene rankings. A final feature importance score was computed by aggregating the results from both datasets as follows, as shown in Equation (4):


(4) \begin{equation*} {\displaystyle \begin{array}{c}\left\{\begin{array}{@{}l}{R}_{\mathrm{all}}={R}_{\mathrm{COVID}-19}+{R}_{\mathrm{CRC}}\\{}{R}_{\mathrm{COVID}-19}=\sum{a}_i\kern5.00em \mathrm{where}\ i,j=1,2,\cdots 5\ \\{}{R}_{\mathrm{CRC}}=\sum{b}_j\end{array}\right.\end{array}} \end{equation*}

Here, ${a}_i$ and ${b}_j$ represent the ranking scores of each gene under the *i*-th and *j*-th feature importance metric in the COVID-19 and CRC models, respectively. Genes were ranked based on the aggregated ${R}_{\mathrm{all}}$ scores, and the top-ranked genes were selected as potential core hub genes for further analysis.

### Single-cell analysis and immune infiltration analysis

To explore the potential molecular mechanisms and regulatory targets of the identified core hub genes, we conducted downstream analyses including single-cell RNA sequencing (scRNA-seq) and immune infiltration analysis. The scRNA-seq analysis enabled high-resolution profiling of gene expression at the single-cell level, allowing us to identify cell-type-specific expression patterns of the hub genes.

To further investigate their immune regulatory roles, we performed immune infiltration analysis, which estimates the relative abundance of 22 immune cell types in bulk RNA-seq data. Correlation analysis revealed specific immune cell types that were positively or negatively associated with hub gene expression, providing complementary evidence of their involvement in the tumor immune microenvironment. Detailed settings for scRNA-seq and immune infiltration analysis are provided in Supplementary Note S3. Together, these analyses provide a multi-faceted view of the functional and immunological relevance of the core hub genes, reinforcing their potential as therapeutic targets.

### Single-gene gene set enrichment analysis

Following the identification of potential core hub genes, single-gene GSEA was conducted for each core hub gene in both groups utilizing the clusterProfiler package. GSEA is a computational method that evaluates whether a predefined collection of genes displays significant, consistent expression differences between two biological conditions or phenotypes [27]. Single-gene GSEA performs association analysis between the target gene and genes in the dataset, identifying a dataset highly correlated with the target gene, which is then used for further analysis [28]. The file was obtained from the MSigDB database for subsequent analysis, and gseaplot2 was used for visualization. The top eight ranked pathway results based on the gene set enrichment scores were plotted for display.

### Scoring function-based microRNA analysis and transcription factor analysis

To investigate the upstream regulatory mechanisms governing the core hub genes, we constructed an integrated TF–miRNA–Target interaction network. TFs and miRNAs are key regulators of gene expression [29] at the transcriptional and post-transcriptional levels, respectively.

Candidate miRNAs targeting the hub genes were retrieved by integrating predictions from the miRcode and miRWalk databases. Potential TFs were identified using the KnockTF database, with log-fold change (logFC) values serving as initial binding scores. The procedures for obtaining miRNAs and TFs are described in Supplementary Note S4. To refine the network, only TFs interacting with at least three core hub genes were retained. The resulting regulatory relationships were visualized using Cytoscape.

To assess the influence of each node (miRNA or TF) within the network, a weighted out-degree centrality (WODC) algorithm was applied. This method considers both the number and the weights of outgoing edges from each node, providing a quantitative measure of its regulatory influence. For miRNAs, the score was calculated as follows, as shown in Equation (5):


(5) \begin{equation*} {\displaystyle \begin{array}{c}{S}_{\mathrm{WODC}}=\sum_{j=1}^n{w}_{ij}\end{array}} \end{equation*}

where ${w}_{ij}$ denotes the weight (absolute logFC) between miRNA *i* and target gene *j*. For TFs, the direct use of logFC values as edge weights introduced limitations, as negative logFC values could be misinterpreted in the centrality calculation. Therefore, an improved scoring function was developed to account for both the magnitude and direction of regulation, as shown in Equation (6):


(6) \begin{equation*} {\displaystyle \begin{array}{c}\left\{\begin{array}{l} Score=\alpha \times{S}_{\mathrm{WODC}}\times symbol\\{}\alpha =\left\{\begin{array}{cc}1,& \left|{S}_c\right|=4\\{}0.5,& \left|{S}_c\right|=3\\{}0.2,& \left|{S}_c\right|=2\\{}0,& \left|{S}_c\right|=0,1\end{array}\right.\\{}{S}_c=\sum_{i=1}^4\operatorname{sign}\left(\log F{C}_i\right)\\{} symbol=\operatorname{sign}\left({S}_c\right)\end{array}\right.\end{array}} \end{equation*}

Here, $symbol$ represents the overall regulatory tendency of a TF across the potential core hub genes, indicating whether it exerts an upregulatory or downregulatory effect. ${S}_c$ reflects the strength of the coordinated effect of the TF on these genes; the larger the value, the stronger the coordinated upregulation or downregulation. $\alpha$ assigns different weights based on the ${S}_c$ value. ${S}_{\mathrm{WODC}}$ represents the result of the weighted out-degree centrality after taking the absolute values of the edges. $Score$ indicates the improved overall score for each TF.

This enhanced scoring function integrates both network topology and biological regulatory consistency to prioritize TFs more accurately with potential upstream regulatory impact on the core hub genes.

### Drug prediction and molecular simulation analysis

To identify potential therapeutics targeting the four core hub genes, we utilized the next-generation LINCS CMap database. LINCS contains large-scale gene expression profiles from human cell lines following small-molecule perturbations, making it a powerful tool for identifying compounds that can reverse disease-associated gene expression signatures. We screened for compounds predicted to reverse the observed core gene signature in cell lines relevant to both COVID-19 and CRC, applying a significance threshold of $P- value<.05$. Next, docking analysis was performed between predicted drugs and target genes. The 3D structures of the predicted drugs were obtained from the PubChem database [30]. The Protein Data Bank (PDB) structures of GPR15, PTGDR2, and FCER1A were obtained from the Research Collaboratory for Structural Bioinformatics (RCSB) PDB database, with PDB IDs 8ZQE, 6D26, and 1F2Q, respectively, while the PDB file for MAL was retrieved from SWISS-MODEL. AutoDock software [31] was used to process both the receptor proteins and ligand small molecules. Details of the simulation setup are described in the Supplementary Note S5. Finally, the complex structure with the lowest binding energy obtained from the molecular docking process was subjected to molecular dynamics simulation using NAMD. The overall structural stability of the complex was analyzed to confirm stable binding, and key interactions and binding modes between the drug and target proteins were elucidated through visualization using PyMOL [32].

## Results

### Differential gene expression and functional enrichment analysis

**Figure 2:**
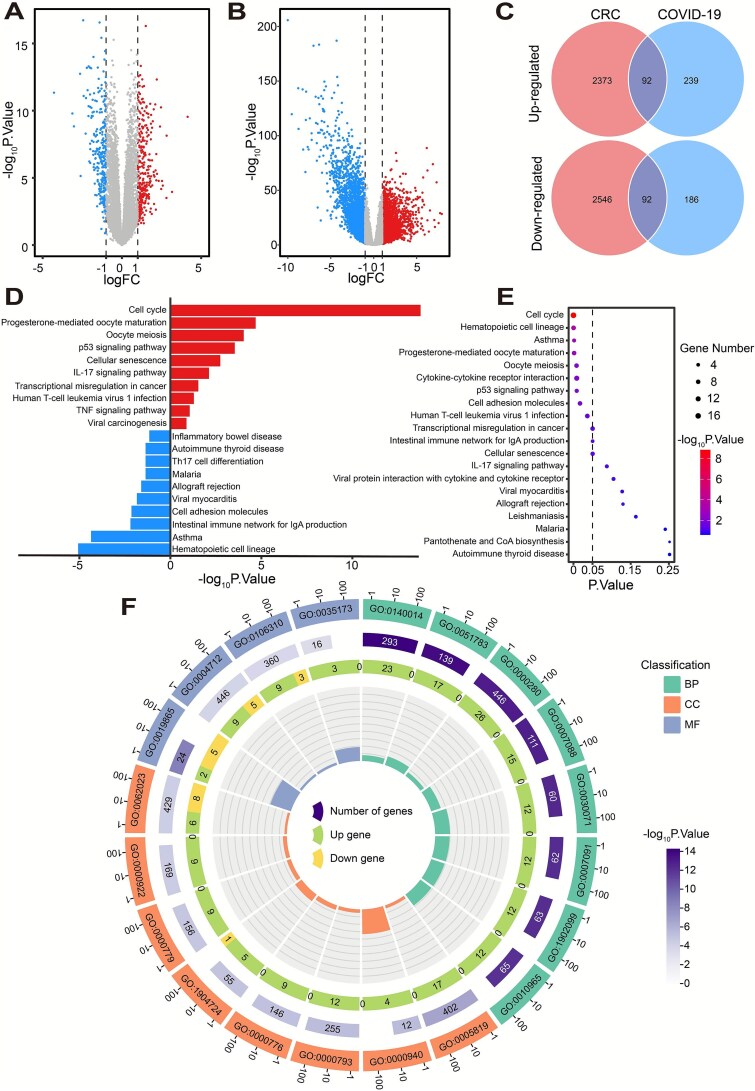
Differential gene identification and enrichment analysis. (A) Volcano plot of differentially expressed genes (DEGs) in COVID-19. (B) Volcano plot of DEGs in CRC. (C) Venn diagram showing commonly upregulated and downregulated DEGs. (D) KEGG enrichment analysis of upregulated and downregulated genes, with the enrichment results for upregulated DEGs distinguished from those for downregulated DEGs by separate groups in the plot. (E) Bubble chart of KEGG enrichment analysis for CDEGs. (F) Circos plot of GO analysis for CDEGs. The outer circle numbers represent GO pathway identifiers, the middle circle numbers represent the number of genes in each GO pathway, and the inner circle numbers represent the number of CDEGs enriched in each pathway, with green indicating upregulated genes.

This study conducted differential gene expression analysis on transcriptome datasets between COVID-19 and CRC (Fig. 2A and B). By intersecting the results of two analyses, a total of 184 CDEGs was identified, including 92 Up-CDEGs and 92 Down-CDEGs (Fig. 2C). Then the pathway analysis was performed on the CDEGs. Initially, we conducted KEGG pathway analysis separately on the 92 upregulated genes and 92 downregulated genes. The results showed that the upregulated genes were primarily enriched in the Cell cycle pathway, while the downregulated genes were mainly enriched in the Hematopoietic cell lineage (Fig. 2D). Subsequently, KEGG and GO analyses were performed on all CDEGs. The KEGG results indicated that a total of 9 pathways demonstrated statistical significance (Fig. 2E). The GO results revealed that the most significantly enriched pathway in BP was Mitotic nuclear division, in CC was Spindle, and in MF was Immunoglobulin binding (Fig. 2F).

### Identification of hub modules by Weighted Gene Co-expression Network Analysis

**Figure 3:**
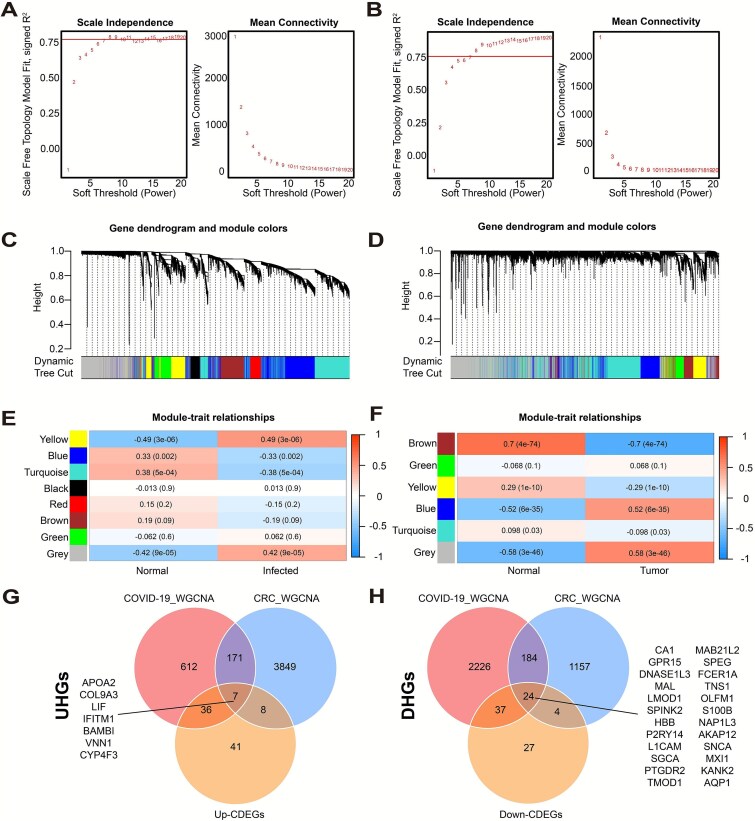
WGCNA reveals shared and distinct modules in COVID-19 and CRC. (A) Determination of soft. Threshold power for COVID-19 genes through scale independence and mean connectivity. The left panels show the relationship between soft-thresholding power and the scale-free topology fit index (*R*^2^). An *R*^2^ > 0.75 indicates the network approximates a scale-free topology. The right panels show mean connectivity, which decreases as power increases. The optimal power is selected as the lowest value achieving scale independence while maintaining sufficient connectivity. (B) Determination of soft threshold power for CRC genes. (C) Gene clustering tree for COVID-19. Each branch represents a gene, and the labels below the dendrogram indicate gene modules (clusters) identified by dynamic tree cutting. Genes in the same module are highly co-expressed. (D) Gene clustering tree for CRC. (E) Correlation heatmap between modules and traits in COVID-19. Module labels on the left correspond to the co-expression modules, and the heatmap cells indicate the strength and direction of the correlation between each module and trait. (F) Correlation heatmap between modules and traits in CRC. (G) Venn diagram for identifying upregulated hub genes (UHGs). (H) Venn diagram for identifying downregulated hub genes (DHGs).

In addition to traditional differential gene expression analysis, WGCNA was performed to identify hub genes. For COVID-19, the soft-thresholding power was set to 8 based on scale independence and mean connectivity (Fig. 3A), and eight gene modules were identified using the TOM matrix with a minimum module size of 300 (Fig. 3C). The yellow module showed the strongest positive correlation with COVID-19 infection (*r* = 0.48, *P* = 3e-08), while the turquoise module showed the strongest negative correlation (*r* = −0.38, *P* = 5e-04) (Fig. 3E). For CRC, the soft-thresholding power was set to 9 (Fig. 3B), resulting in six gene modules. The gray module had the strongest positive correlation with tumor occurrence (*r* = 0.58, *P* = 3e-46), and the brown module had the strongest negative correlation (*r* = −0.70, *P* = 4e-74) (Fig. 3D and F).

These high correlation values reflect strong co-expression patterns and consistent gene expression changes across samples, indicating that these modules are likely enriched with genes functionally related to disease progression. In particular, highly positive correlations may indicate modules where genes are strongly upregulated in disease conditions, while highly negative correlations suggest modules where genes are strongly downregulated—both reflecting robust and disease-relevant transcriptional programs. Finally, the positively correlated genes were intersected with the previously identified Up-CDEGs (Fig. 3G), and the negatively correlated genes were intersected with the Down-CDEGs (Fig. 3H), resulting in 7 upregulated hub genes (UHGs) and 24 downregulated hub genes (DHGs), for a total of 31 hub genes.

### Identification of potential core hub genes based on machine learning algorithms

**Figure 4:**
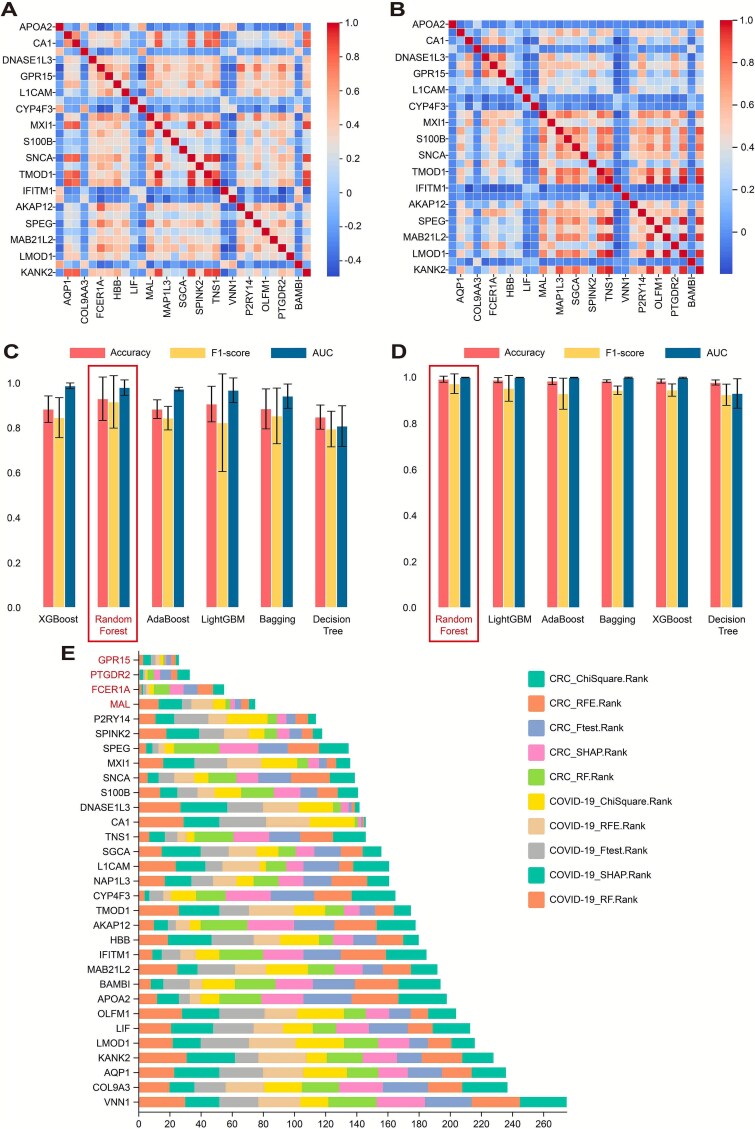
Machine learning selection of core hub genes. (A) Correlation heatmap of 31 hub genes from COVID-19. (B) Correlation heatmap of hub genes from CRC. (C) Machine learning model selection for hub genes in COVID-19. The model is evaluated using three metrics: Accuracy, F1-score, and AUC. (D) Machine learning model selection for hub genes in CRC. (E) Integrating the results of the COVID-19 and CRC machine learning models, feature importance is assessed using multiple selection methods. Genes with the smallest cumulative sum of feature importance are considered the most important.

Based on the methodology described above, machine learning models were trained and evaluated using COVID-19 and CRC datasets. The Random Forest model achieved superior performance in both datasets. For the COVID-19 model, the accuracy, F1-score, and AUC were 0.930, 0.916, and 0.980, respectively. For the CRC model, the corresponding values were 0.992, 0.972, and 0.999 (Figs 3D and 4C).

Following model training, feature importance rankings were generated (Tables S2 and S3), and the top genes were identified based on their final aggregated scores. The top four genes—GPR15, PTGDR2, FCER1A, and MAL—were selected as potential core hub genes. Their differential expression in both cohorts is shown in Fig. S1, and all differences were statistically significant.

### Validation of the functional importance of core hub genes in predictive modeling

**Figure 5:**
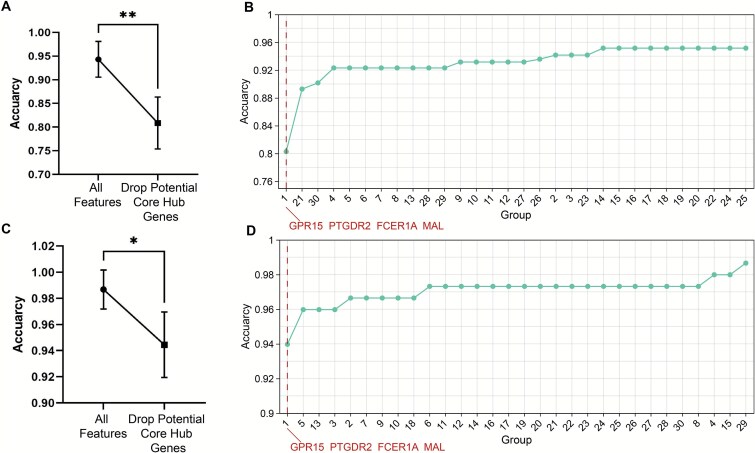
Evaluation of the predictive contribution of potential core hub genes. (A) Classification accuracy of the random forest model on the COVID-19 dataset before and after removing the potential core hub genes, as assessed using a *t*-test (*P* = .008). (B) Group-wise feature exclusion analysis in the COVID-19 dataset. Thirty gene groups with four randomly selected genes each were excluded one by one, along with a separate group containing the potential core hub genes (GPR15, PTGDR2, FCER1A, and MAL). The *x*-axis represents the index of each of the 30 random feature exclusion experiments, with the specific genes excluded in each group detailed in Tables S4 and S5. The *y*-axis indicates the classification accuracy of the model after each exclusion, reflecting the impact of each gene group on predictive performance. The results are sorted in ascending order of model accuracy to facilitate comparison across different gene exclusion scenarios. (C) Classification accuracy of the random forest model on the CRC dataset before and after removing the potential core hub genes, as assessed using a *t*-test (*P* = .041). D) Group-wise feature exclusion analysis in the COVID-19 dataset.

To validate the scientific significance of the identified potential core hub genes in the predictive model, we designed two random forest–based machine learning validation strategies. In these models, the input features consisted of the remaining 31 original features after feature selection and elimination, while the output was a binary classification label indicating disease status (healthy versus diseased). First, we compared the classification performance of the model with and without the inclusion of these potential core hub genes. As shown in Fig. 5A and C, the removal of these genes resulted in a decrease in prediction accuracy in both datasets, and this decrease was statistically significant, indicating that these genes play a critical role in classification performance.

Second, we iteratively excluded 30 different gene subsets, each consisting of four genes randomly selected from the pool of 31 candidate genes. The details of the random gene selection are provided in Tables S4 and S5. The potential core hub genes were grouped separately and evaluated as an independent subset. Model performance was monitored after each exclusion to assess the predictive contribution of each group. Notably, exclusion of the group containing GPR15, PTGDR2, FCER1A, and MAL resulted in the most significant drop in accuracy (Fig. 5B and D), further supporting their central role in model prediction.

Collectively, these results highlight the robustness and biological relevance of the selected core hub genes, confirming their central position in both gene co-expression networks and predictive modeling, and underscoring their scientific importance.

### Expression of potential core hub genes in single cells

**Figure 6:**
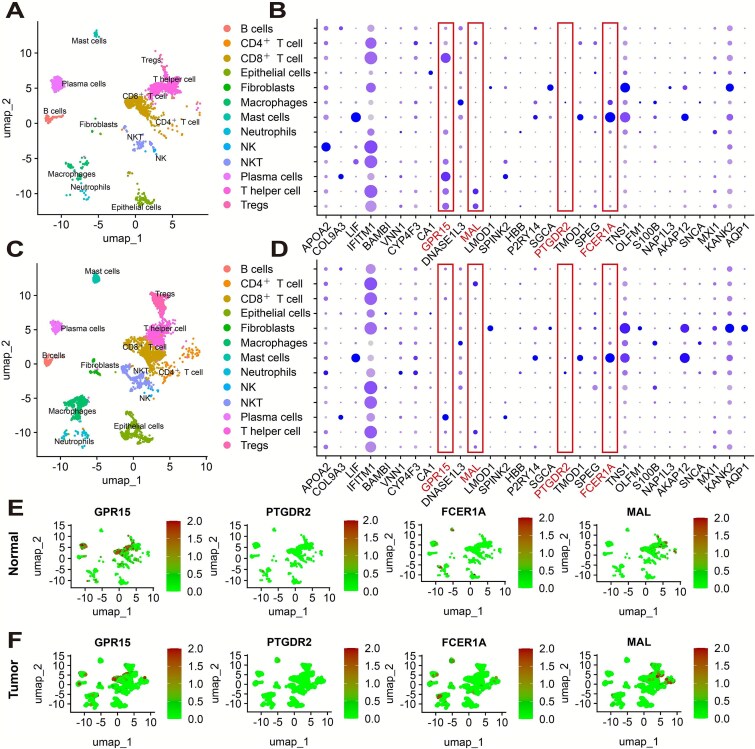
Gene expression in single cells. (A) Cell annotation in normal tissue using UMAP clustering. Each dot represents a single cell, distinguished by cell type. (B) Expression of 31 hub genes in normal tissue. The intensity of the circles reflects the average expression level, and the circle size reflects the percentage of cells expressing each gene. Selected genes are identified as potential core hub genes. (C) Cell annotation in tumor tissue. (D) Expression of 31 hub genes in tumor tissue. (E) Distribution of expression levels of the four potential core hub genes in normal tissue. Higher expression levels are represented by greater signal intensity, whereas lower expression levels are represented by weaker signal intensity. Each panel corresponds to one gene and shows its expression pattern across single cells, allowing visualization of gene expression within specific cell subpopulations. (F) Distribution of expression levels of the four potential core hub genes in tumor tissue. Notably, a comparison with normal tissue indicates a general downregulation of these genes in malignant samples.

Single-cell analysis was performed using the GSE146771 dataset with the Seurat package. Clustering was conducted using Uniform Manifold Approximation and Projection (UMAP), with the dims parameter optimized to 35 based on performance across six tested values (Fig. S2), and the resolution parameter set to 0.2 for downstream analysis (Fig. S3). Differentially expressed genes for each cluster were identified using the FindMarkers function and used for single-cell annotation. Normal and tumor groups were extracted separately, and cells were classified into 13 clusters, including CD8^+^ T cells (Fig. 6A and C). A DotPlot was generated to visualize the expression patterns of the 31 hub genes across cell types. These genes also served as input features for machine learning. Differences in expression between normal and tumor cells were observed, particularly in CD8^+^ T cells, natural killer (NK) cells, natural killer T (NKT) cells, and plasma cells (Fig. 6B and D).

Further analysis focused on the four core hub genes (GPR15, PTGDR2, MAL, FCER1A) in normal versus tumor groups (Fig. 6E and F). GPR15 showed marked downregulation in tumors, while the other three genes also exhibited varying degrees of reduced expression. VlnPlots (Fig. S4) revealed that GPR15 and MAL were highly expressed in T cells, FCER1A was enriched in mast cells, and PTGDR2 showed weak but specific expression in eosinophil-related populations. These patterns suggest distinct roles for these genes in immune cell subsets within the CRC microenvironment.

### Immune infiltration analysis of potential core hub genes

**Figure 7:**
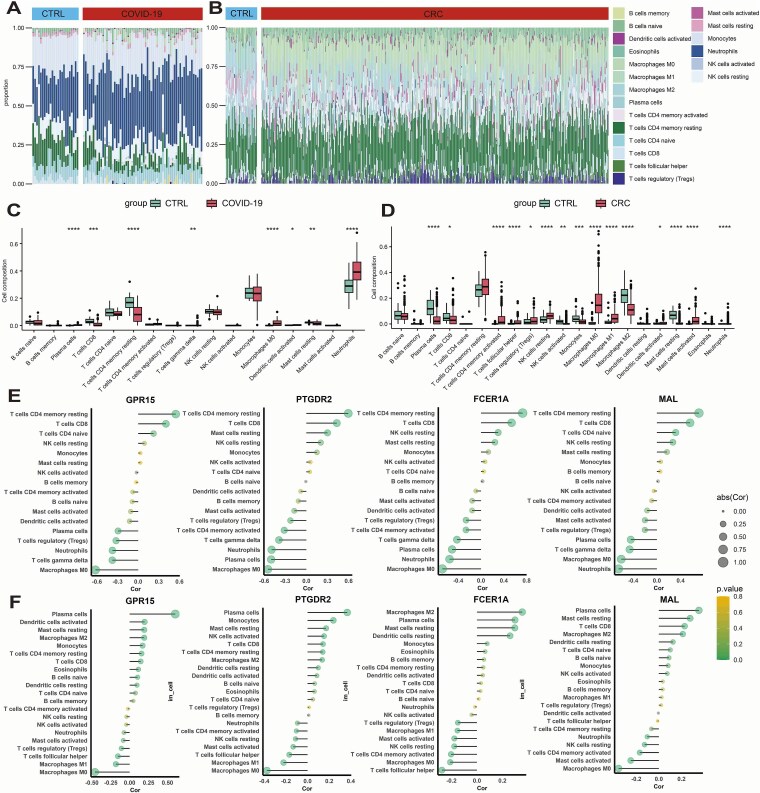
Immune infiltration analysis results. (A) Stacked bar plots showing the immune infiltration landscape of 22 immune cell types in individual samples from the COVID-19 datasets. Each bar represents one sample, and the proportion of each immune cell type is indicated by a distinct pattern or legend entry. Disease samples and healthy controls are distinguished by separate group labels in the header. (B) Stacked bar plot of immune cell infiltration in the CRC dataset. (C) Boxplots comparing the relative abundance of each immune cell type between disease and control groups in COVID-19. Each dot represents a sample. Asterisks indicate significance levels (*P* < .05, *P* < .01, *P* < .001, *P* < .0001). (D) Boxplot showing the differential abundance of various immune cell types between the CRC and normal control groups. (E) Correlation between the expression of potential core hub genes and immune cell infiltration in the COVID-19 group, visualized using a lollipop plot. The *x*-axis indicates the Cor, with positive values showing positive correlation and negative values showing inverse correlation. The size of each dot reflects the abs(Cor): the larger the dot, the stronger the correlation. The visual encoding of the dots also reflects the P‑value, with more prominent markers indicating higher statistical significance. (F) Correlation between the expression of potential core hub genes and immune cell infiltration in the CRC group, visualized using a lollipop plot.

In the single-cell analysis of the CRC dataset, the four potential core hub genes showed strong correlations with various immune cell types. To further explore their roles within the immune microenvironment, immune infiltration analysis was performed. CIBERSORT output was normalized to 1 for each sample to improve the interpretability of the stacked bar plots (Fig. 7A and B). A *T*-test was used to compare immune cell proportions between normal and tumor groups, revealing shared alterations in immune cell composition across both the COVID-19 and CRC datasets (Fig. 7C and D).

For gene–immune cell correlation analysis, a single-sample Kolmogorov–Smirnov test was first used to assess data normality. For non-normally distributed data, Spearman correlation coefficients and *P*-values were calculated and visualized using lollipop plots, highlighting the direction and strength of associations (Fig. 7E and F). Notably, neutrophils, macrophages M0, and regulatory T cells (Tregs) were negatively correlated with gene expression, while resting mast cells and CD8^+^ T cells showed positive correlations.

Given that these four core hub genes were downregulated, the results suggest that their decreased expression may reduce infiltration of CD8^+^ T cells and resting mast cells, potentially impairing antitumor immunity. Meanwhile, increased infiltration of immunosuppressive cells such as neutrophils, macrophages M0, and Tregs could promote tumor immune escape and facilitate CRC progression.

### Single-gene gene set enrichment analysis of potential core hub genes

**Figure 8:**
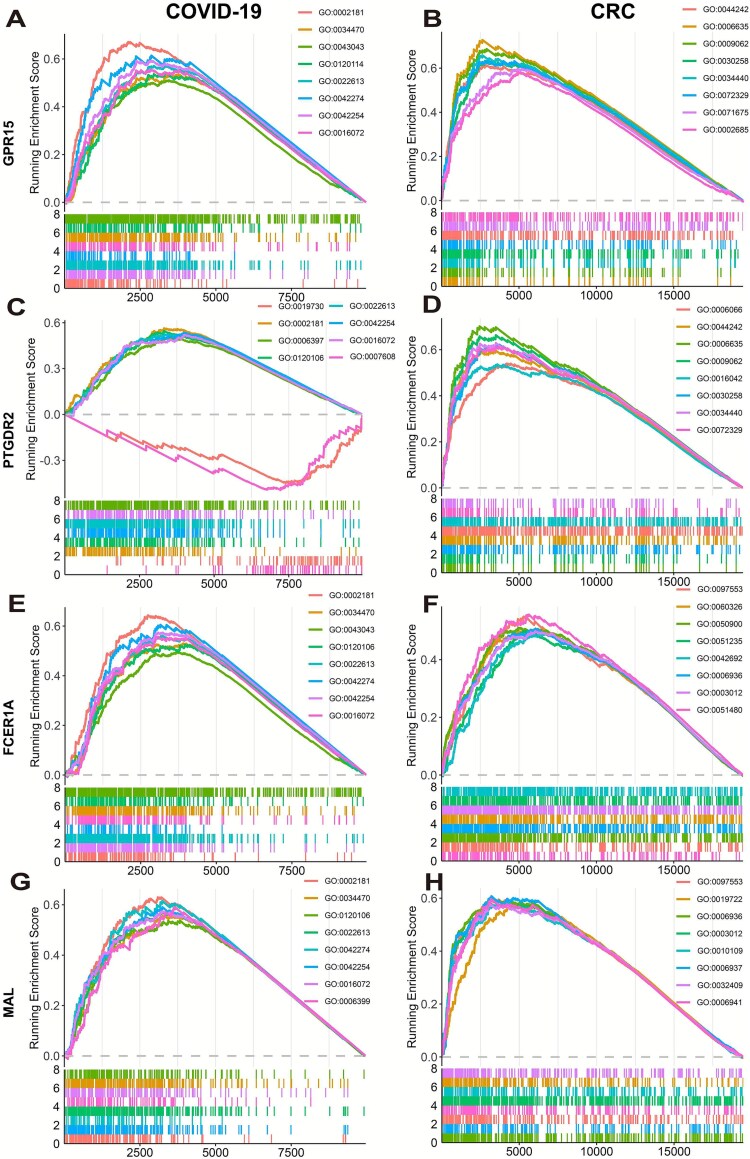
Single-gene GSEA analysis results. Each panel presents the GSEA enrichment plots for the top 10 significantly enriched GO-BP terms associated with a specific gene in two disease contexts. The *x*-axis shows the gene rank in the ordered dataset, while the *y*-axis represents the running enrichment score. The vertical lines in the middle indicate the positions of genes from each GO term in the ranked gene list. (A, B) GSEA analysis for GPR15 in COVID-19 and CRC. Enriched in ribosome-related and translation-associated pathways, suggesting their involvement in ribosomal protein synthesis and translational control. (C, D) GSEA analysis for PTGDR2 in COVID-19 and CRC. Identified GO terms are primarily associated with prostaglandin signaling, inflammatory response, and G protein–coupled receptor activity, indicating a potential role in inflammation-mediated pathways. (E, F) GSEA analysis for FCER1A in COVID-19 and CRC. Enriched in pathways related to calcium ion transmembrane transport. G, H) GSEA analysis for MAL in COVID-19 and CRC. Enriched in pathways related to RNA metabolic processes and fatty acid metabolism.

Single-gene GSEA was performed on the core hub genes identified via machine learning, with results summarized in Fig. 8. In the COVID-19 dataset, these genes were significantly enriched in ribosome-related pathways, highlighting their role in protein translation. In the CRC dataset, they were mainly associated with calcium ion transport, RNA and fatty acid metabolism, and muscle system processes. Notably, previous studies have shown that intracellular Ca^2+^ levels can influence T-cell protein translation and thus modulate immune function [33, 34].

Despite differences, both datasets showed shared enrichment in translation, RNA, and fatty acid metabolism pathways, particularly those related to cytoplasmic translation and RNA processing. These findings suggest that dysregulation in transcription and translation may contribute to disease progression and severity.

### Scoring function-based identification of relevant microRNA and transcription factor

**Figure 9:**
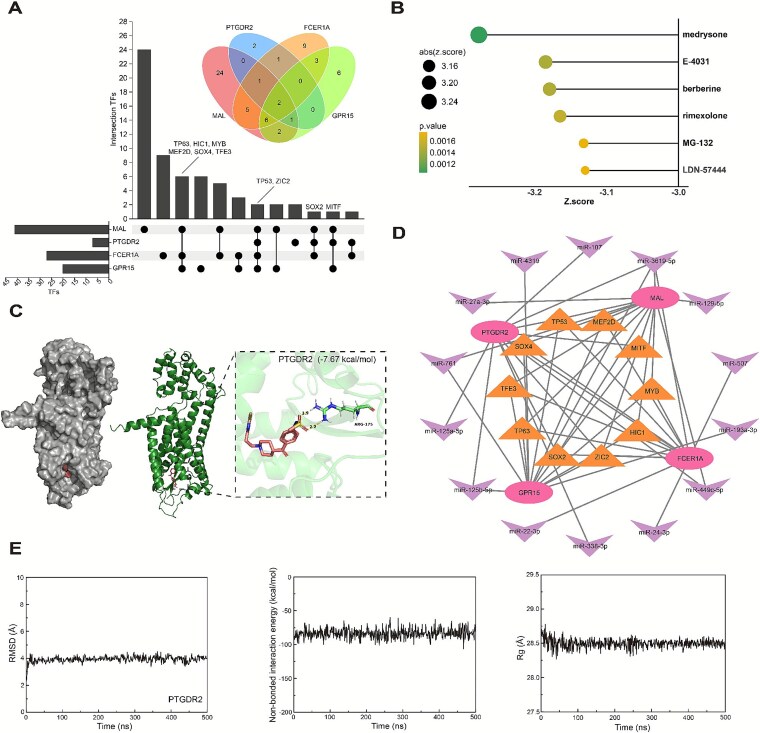
TF–miRNA–Target network construction and drug prediction. (A) Upset plot of TFs associated with GPR15, PTGDR2, FCER1A, and MAL. The horizontal bars represent the total number of TFs interacting with each gene. The vertical bars indicate the number of TFs shared across different gene combinations, as marked by the dots below each bar. A Venn diagram highlights overlapping TFs among the four genes. (B) Drugs related to CRC or COVID-19 predicted by LINCS, visualized using a lollipop plot. The *Z*-score reflects the potential of each drug to modulate gene expression in a direction opposite to the disease signature. Dot size represents the absolute *Z*-score, and the visual emphasis of the dots reflects the P-value, with more prominent markers indicating higher statistical significance. (C) TF–miRNA–Target interaction network. Distinct node shapes or legend categories are used to differentiate miRNAs, potential core hub genes, and TFs. (D) Docking results of E-4031 with PTGDR2. The docking pose of E-4031 is highlighted in the binding pocket, with a binding affinity of – 7.67 kcal/mol, suggesting a stable interaction. (E) Molecular dynamics simulation results for E-4031 with PTGDR2.

Based on the scoring strategy described in the Materials and Methods section, a TF–miRNA–Target interaction network was successfully constructed. Integration of miRcode and miRWalk databases identified a set of miRNAs targeting the core hub genes, while TFs regulating at least three hub genes were retained using KnockTF data (Fig. 9A).

The weighted out-degree centrality algorithm was applied to quantitatively assess the regulatory influence of miRNAs and TFs. For miRNAs, miR-3619-5p received the highest score, suggesting a strong regulatory potential across the hub genes (Table S6). In the TF module, the use of an improved scoring function allowed for direction-sensitive evaluation. Results showed that p53 had a high positive score (Table S7), indicating that its knockout significantly increased the expression of hub genes. Similarly, MYB and ZIC2 demonstrated moderate upregulation effects, while MITF exhibited a negative score, implying a potential repressive function (Fig. 9D).

These findings highlight several upstream regulatory elements with potential functional relevance and therapeutic value in modulating the expression of core hub genes.

### Identification of potential drugs targeting potential core hub genes

The LINCS database was utilized to predict all potential drugs targeting GPR15, PTGDR2, FCER1A, and MAL. Six candidate drugs were identified: medrysone, E-4031, berberine, rimexolone, MG-132, and LDN-57444 (Fig. 9B). Molecular docking of these receptor proteins with the selected small-molecule ligands was performed using AutoDock to evaluate the potential of these drugs to bind to these proteins. The docking results, summarized in Table S8, demonstrated that medrysone, E-4031, and rimexolone exhibited stable, high-affinity binding to all four proteins. Hydrogen bonding interactions at the binding sites were visualized using PyMOL, revealing that E-4031 consistently formed stable hydrogen bonds with key residues of all four proteins (Fig. 9C and Fig. S5A, D, and  G). Taking the interaction between PTGDR2 and E-4031 as an example, the ligand molecule forms two stable hydrogen bonds within the binding pocket of the protein, interacting with the side chain of the key residue ARG-175 (arginine). The hydrogen bond distances are 1.9 and 2.2 Å, respectively, and the docking binding energy is −7.67 kcal/mol, indicating strong binding stability (Fig. 9C).

The molecular dynamics simulation was subsequently applied to analyze the binding interaction between all four proteins and E-4031. The simulation was performed for 500 ns to ensure system stability and reliable interaction analysis. The molecular dynamics simulation results of the interactions between the four proteins and E-4031 are shown in Fig. 9E and Fig. S5B, D, and  F. Among them, the binding of PTGDR2 with E-4031 was the most stable (Fig. 9E). As shown in Fig. 9E, the root mean square deviation (RMSD) values of the protein–ligand complex quickly reached equilibrium within the initial 50 ns and remained consistently stable around 2.5 Å throughout the entire simulation, indicating minimal structural fluctuations and a well-converged system. The non-bonded interaction energy fluctuated within a narrow range around −100 kcal/mol, suggesting a stable and energetically favorable binding interaction with no significant disruption over time. Importantly, the Rg values remained steady at ~28.5 Å, reflecting the compactness and structural integrity of the PTGDR2–E-4031 complex during the simulation. Based on these findings, E-4031 is suggested as a promising drug candidate with the potential to target the core hub genes implicated in both COVID-19 and CRC.

## Discussion

In this study, we employed an integrative bioinformatics pipeline to identify key immune-related hub genes associated with both COVID-19 and CRC. Through differential gene expression analysis and WGCNA, we identified 7 upregulated and 24 downregulated hub genes. WGCNA enabled the detection of co-expressed gene modules correlated with disease traits, capturing systemic gene regulation patterns beyond individual gene changes. To further refine our gene list, we applied machine learning–based feature selection algorithms. These methods are well suited for high-dimensional transcriptomic data and help eliminate redundancy while identifying robust predictors. This approach ultimately narrowed down the set of candidates to four potential core hub genes: GPR15, PTGDR2, FCER1A, and MAL. All four genes are closely linked to immune responses and inflammatory signaling. While prior studies have reported their roles in various immune-related and cancer contexts [35–40], our results provide new evidence that these genes are consistently downregulated in both COVID-19 and CRC. To further evaluate their scientific relevance, we additionally validated their functional importance in predictive modeling.

To further explore the cellular context of these findings, we performed single-cell transcriptomic analysis and immune infiltration analysis. Single-cell analysis revealed significantly lower expression of immune cell types such as CD8^+^ T cells, NK cells, NKT cells, and plasma cells in tumor tissues compared to normal tissues. Complementary immune infiltration analysis showed a consistent trend across both diseases: neutrophils, M0 macrophages, and Tregs were significantly increased, while resting mast cells and CD8^+^ T cells were markedly reduced. These findings are consistent with previous studies reporting immune suppression in cancer and severe COVID-19 cases [41–44].

To investigate the regulatory mechanisms underlying the observed gene expression changes, we conducted single-gene GSEA, which revealed that the four core hub genes are enriched in overlapping pathways related to RNA metabolism, translation, and fatty acid metabolism. These pathways highlight potential disruptions in transcriptional and translational processes. To elucidate upstream regulators, we constructed a TF–miRNA–Target interaction network and introduced an improved weighted out-degree centrality scoring algorithm to prioritize key regulators. This method quantitatively evaluates the influence of each TF and miRNA based on the number and strength of their downstream interactions, offering an interpretable metric of regulatory importance. From this analysis, miR-3619-5p and p53 emerged as top regulators. Previous studies suggest that miR-3619-5p can stabilize mRNA in CD8^+^ T cells by preventing degradation [45], thereby enhancing protein translation. On the other hand, p53, typically a tumor suppressor [46], has been shown in some contexts to suppress T-cell activity, particularly CD8^+^ T cells, potentially impairing antitumor immunity [47]. Our results suggest that upregulation of p53 and dysregulation of miR-3619-5p may contribute to the impaired transcriptional and translational capacity of CD8^+^ T cells in both diseases.

Finally, drug prediction analysis using the LINCS database identified E-4031, a selective hERG potassium channel blocker [48], as a potential therapeutic candidate capable of reversing the expression patterns of the identified hub genes. Although E-4031 is primarily used to treat arrhythmia [49], its interaction with PTGDR2 was confirmed through molecular docking and molecular dynamics simulations, demonstrating stable binding affinity and dynamic stability. Few studies have explored the potential of E-4031 as a therapeutic agent for CRC or COVID-19. Our findings suggest that E-4031 may merit further investigation through experimental validation.

However, this study has limitations. The datasets used were relatively outdated and limited in size, which may affect data reliability. Moreover, while molecular docking and dynamics simulations supported the interaction between E-4031 and the target proteins, no *in vitro* or clinical validation was conducted to confirm therapeutic efficacy.

In conclusion, this study establishes an integrative analytical framework that combines differential expression analysis, WGCNA, and machine learning–based feature selection to identify shared hub genes in COVID-19 and CRC. By integrating bulk transcriptomic data with single-cell–level dissection of immune cell subsets, we not only pinpoint core genes but also clarify their predominant cellular contexts. Furthermore, we couple these cross-disease signatures to drug repositioning by confirming candidate drug–target interactions with molecular docking followed by molecular dynamics simulations. Together, these innovations provide a refined view of immune dysregulation common to both diseases and offer testable hypotheses and methodological guidance for future mechanistic and translational studies on CRC–COVID-19 comorbidity.

## Conclusion

In this study, bioinformatics and machine learning approaches identified GPR15, PTGDR2, FCER1A, and MAL as potential core hub genes and diagnostic markers shared by both COVID-19 and CRC. Further analysis suggested that dysregulation of TF p53 (upregulated) and miR-3619-5p (downregulated) may drive disease progression by impairing transcription and translation, leading to reduced expression of these core genes and weakening CD8^+^ T-cell immune infiltration. This immune suppression also indirectly affects other cells like M0 macrophages. Additionally, E-4031 was identified as a potential therapeutic agent targeting all four hub genes. These findings provide new insights into the shared immune dysregulation mechanisms in COVID-19 and colorectal cancer, identify potential therapeutic targets and candidate drugs, and offer novel directions for future treatment strategies.

**Key Points**

- Integration of bioinformatics and machine learning approaches enables efficient screening of diagnostic biomarkers for concurrent COVID-19 and colorectal cancer (CRC).
- Multi-dimensional analyses including single-cell sequencing, immune infiltration, and regulatory network construction reveal molecular mechanisms underlying the comorbidity between COVID-19 and CRC.
- Mathematical modeling of TF–miRNA–target networks quantifies the regulatory roles of each component, identifying key molecular drivers of disease progression.
- Drug screening and molecular dynamics simulations identify potential therapeutic compounds with optimal binding to diagnostic targets.

## Supplementary Material

Supplementary_Material_bbag065

## Data Availability

The data underlying this article are available in the public domain. The transcriptomic datasets analyzed in this study were obtained from GEO under the accession numbers GSE152641 and GSE146771 (https://www.ncbi.nlm.nih.gov/geo/), and from TCGA-COAD via the UCSC Xena (https://xenabrowser.net/datapages/). miRNA-target interaction data were derived from the miRcode database (http://www.mircode.org/), miRWalk database (http://mirwalk.umm.uni-heidelberg.de/), and transcription factor regulation data were obtained from the KnockTF database (http://www.licpathway.net/KnockTF). Protein–protein interaction data were retrieved from the STRING database (https://cn.string-db.org/). All datasets are publicly accessible, and no new raw sequencing data were generated in this study.
