# Supplementary material for: Revealing shared molecular markers and mechanisms in colorectal cancer and COVID-19 through bioinformatics and machine learning
Source: Brief Bioinform. 2026 Feb 23;27(1):bbag065. doi: 10.1093/bib/bbag065 (PMC12927884; doi:10.1093/bib/bbag065)
Supplement: Supplementary_Material_bbag065 [file supplementary_material_bbag065.docx]

Supplementary data

Revealing shared molecular markers and mechanisms in colorectal cancer and COVID-19 through bioinformatics and machine learning

Hairui Wang*a,b*,1, Haodong Yao*b,c*,1, Wenchao Niu*b*, Rongchang Xing*a,b* , Lu Cai*a,b*, Zuoxin Xi*b,d*, Shichen Gao*^a^* and Lina Zhao*b,c*,*

*a School of Science, China University of Geosciences, Beijing, Xueyuan Road 29, Haidian District, 100083, Beijing, China*

*b Multi-disciplinary Research Division, Institute of High Energy Physics, Chinese Academy of Sciences, 19B Yuquan Road, Shijingshan District, 100049, Beijing, China*

*c University of Chinese Academy of Sciences, 19A Yuquan Road, Shijingshan District, 100049, Beijing, China*

*d School of Information Engineering, Minzu University of China, 27 Zhongguancun South Avenue, 100083, Beijing, China*

* Corresponding author: linazhao@ihep.ac.cn

1 These authors contributed equally to this work.

Supplemental Notes, Tabels and Figures

List of Notes

**Note 1. Differential gene expression analysis setup 3**

**Note 2. Machine learning training data processing and model selection 3**

**Note 3. Details of scRNA-seq and immune infiltration analysis 3**

**Note 4. Acquisition of microRNAs and transcription factors. 3**

**Note 5. Molecular simluation setup 4**

List of Tables

**Table 1. Summary of public transcriptomic datasets used in this study. 5**

**Table 2. Scores of the top ten ranked feature genes for COVID-19 5**

**Table 3. Scores of the top ten ranked feature genes for CRC 5**

**Table 4. Random feature exclusion analysis for COVID-19 classification: detailed groups of four-gene subsets 6**

**Table 5. Random feature exclusion analysis for CRC classification: detailed groups of four-gene subsets 7**

**Table 6. Scores of MicroRNAs (miRNAs) 10**

**Table 7. Scores of transcription factors (TFs) 10**

**Table 8. Binding Energies between small molecule drug and target protein 11**

List of Figures

**Figure 1. Box plots of potential core hub gene expression in two datasets. 8**

**Figure 2. The UMAP clustering results under different dims parameters, with dims set to 15, 20, 25, 30, 35, and 40. 8**

**Figure 3. The cell clustering and lineage trajectory maps under different resolution parameters, with different colors representing different resolutions. 9**

**Figure 4. The violin plots showing the distribution of different genes across various cell types. 9**

**Figure 5. GPR15, FCER1A, and MAL docking scores and molecular dynamics simulations. 11**

Notes

1. **Differential gene expression analysis setup**

The limma package [1] was used to compare the normal and disease-affected groups, and the screening criteria for differentially expressed genes (DEGs) were P.value<0.05 and |logFC|>1. Up-regulated and down-regulated DEGs were identified separately for each dataset. The intersection of these DEGs was then determined to obtain common differentially expressed genes (CDEGs) between the two datasets.

Pathway enrichment analysis and visualization were performed using the clusterProfiler R package [2]. Separate KEGG pathway analyses were conducted on up-regulated and down-regulated genes within the common DEGs. Statistical significance for enrichment was established using criteria of overlap≥3 and P.value<0.05. The KEGG results were visualized using bar plots and bubble plots, while GO results were illustrated using Circos plots generated through ChiPlot (<https://www.chiplot.online/>).

1. **Machine learning training data processing and model selection**

Two separate machine learning models were trained for COVID-19 and CRC datasets. For the COVID-19 data, a matrix of hub genes was used as input features (86 genes), with patient samples as rows and disease status as labels, resulting in an 86 × 31 feature matrix and a corresponding label vector. A similar approach was applied to the CRC dataset, resulting in a 494 × 31 feature matrix. Six machine learning algorithms were employed for model training and comparison. Model performance was evaluated using cross-validation, and three commonly used evaluation metrics—accuracy, F1-score, and AUC—were used to select the optimal model. The Random Forest algorithm demonstrated the best performance across both datasets and was therefore selected for downstream interpretability analysis.

1. **Details of scRNA-seq and immune infiltration analysis**

For single-cell transcriptomic analysis, the Seurat package (v4.0) was used [3]. Cells were clustered using the UMAP algorithm, and differential expression between clusters was identified using the FindAllMarkers function. Marker genes were ranked by avg_log2FC, and the top 20 genes were selected for cell-type annotation. The advantage of scRNA-seq lies in its ability to resolve cell-type-specific expression, avoiding the averaging effect seen in bulk transcriptomics [4].

Immune infiltration analysis was conducted using the CIBERSORT algorithm with the LM22 gene signature [5]. Spearman correlation analysis was performed between hub gene expression levels and the inferred proportions of immune cell types. Correlation coefficients and p-values were used to identify statistically significant associations.

1. **Acquisition of microRNAs and transcription factors**

Increasing evidence suggests that miRNAs can serve as potential biomarkers for disease [6], and dynamic changes in TF networks significantly influence complex biological processes, such as cancer progression and immune regulation [7]. In this study, the miRcode database (http://www.mircode.org/) [8] and the miRWalk database (http://mirwalk.umm.uni-heidelberg.de/) [9] were used to obtain miRNAs and the binding scores related to potential core hub genes. These databases integrate both predictive and experimentally validated interactions across multiple species, providing comprehensive and high-coverage datasets that are widely used in studies of non-coding RNA regulatory mechanisms.

To investigate the regulatory interactions between TFs and the four candidate genes, we employed the KnockTF database (http://www.licpathway.net/KnockTF) [10]. KnockTF contains manually curated RNA-seq and microarray datasets from TF/TcoF knockdown or knockout experiments across various species and tissue types. It is particularly suitable for identifying regulatory factors associated with specific genes and exploring their functional implications.

Finally, protein-protein interaction (PPI) data for the four target genes were retrieved from the STRING database (https://cn.string-db.org/) [11]. As a widely used resource, STRING integrates known and predicted protein interactions with confidence scoring, making it ideal for constructing high-quality interaction networks and analyzing functional connectivity among proteins.

1. **Molecular simluation setup**

The grid box position and size were set based on commonly observed binding sites of the proteins in experimental studies. Molecular docking was then performed to calculate the binding affinity between the receptor proteins and ligand molecules. Among the 50 conformations searched by the genetic algorithm, the one with the lowest binding energy was selected as the final result. The binding threshold was set at -5 kcal/mol, with values below this threshold considered indicative of stable binding.

To further investigate the stability of the docked complexes, molecular dynamics (MD) simulations were performed under the NPT ensemble with periodic boundary conditions in all directions. The systems were equilibrated using the Langevin thermostat and the Nosé–Hoover Langevin piston method under standard conditions. Electrostatic interactions were calculated using the particle mesh Ewald (PME) method under conducting boundary conditions, and van der Waals interactions were truncated at 12 Å. The CHARMM22 and CHARMM36 force fields were used to describe the proteins, while the TIP3P model was applied for explicit water molecules. Simulations were carried out using NAMD 3.0. Initial equilibration steps included 50,000 steps of steepest descent energy minimization, followed by gradual heating to 300 K over 20 ns. Temperature coupling and ion balance were achieved by introducing a sodium ion solution during the equilibration phase.

Table 1. Summary of public transcriptomic datasets used in this study.

| Dataset ID | Data Type | Platform | Sample Description | Application in Study |
| --- | --- | --- | --- | --- |
| GSE152641 | Bulk  RNA-seq | Illumina | 24 healthy controls,  62 COVID-19 patients | COVID-19  transcriptome data |
| TCGA-COAD | Bulk  RNA-seq | Illumina (TCGA pipeline) | 41 adjacent normal,  453 CRC tissues | CRC transcriptome data |
| GSE146771 | Single-cell RNA-seq | SMART-seq2 | scRNA-seq from 10  CRC patients | Single-cell validation |

Table 2. Scores of the top ten ranked feature genes for COVID-19

| Feature | RF | SHAP | Ftest | Chisquare | RFE | R_COVID-19_ |
| --- | --- | --- | --- | --- | --- | --- |
| PTGDR2 | 0.146 | 0.060 | 89.608 | 9.102 | 1 | 6 |
| FCER1A | 0.115 | 0.068 | 72.314 | 8.051 | 2 | 10 |
| GPR15 | 0.094 | 0.032 | 53.022 | 8.101 | 3 | 16 |
| SPEG | 0.081 | 0.040 | 40.364 | 3.697 | 4 | 23 |
| TNS1 | 0.055 | 0.010 | 28.704 | 4.077 | 6 | 36 |
| CYP4F3 | 0.088 | 0.057 | 25.101 | 1.985 | 5 | 37 |
| AKAP12 | 0.037 | 0.014 | 35.103 | 3.686 | 9 | 40 |
| SNCA | 0.059 | 0.018 | 24.120 | 2.862 | 13 | 45 |
| APOA2 | 0.032 | 0.004 | 32.348 | 2.549 | 7 | 52 |
| IFITM1 | 0.043 | 0.030 | 18.826 | 2.218 | 10 | 52 |

**Table 3. Scores of the top ten ranked feature genes for CRC**

| Feature | RF | SHAP | Ftest | Chisquare | RFE | R_CRC_ |
| --- | --- | --- | --- | --- | --- | --- |
| CA1 | 0.125 | 0.008 | 1023.975 | 146.647 | 1 | 7 |
| GPR15 | 0.161 | 0.013 | 774.472 | 87.039 | 3 | 10 |
| DNASE1L3 | 0.096 | 0.007 | 935.104 | 81.533 | 2 | 17 |
| MAL | 0.112 | 0.004 | 638.538 | 52.729 | 5 | 19 |
| PTGDR2 | 0.097 | 0.007 | 431.628 | 36.892 | 4 | 27 |
| P2RY14 | 0.081 | 0.006 | 433.050 | 44.173 | 8 | 31 |
| MXI1 | 0.080 | 0.006 | 503.480 | 29.503 | 6 | 34 |
| SPINK2 | 0.051 | 0.005 | 295.634 | 41.290 | 7 | 37 |
| FCER1A | 0.024 | 0.003 | 281.566 | 37.304 | 10 | 45 |
| TMOD1 | 0.016 | 0.003 | 199.772 | 16.727 | 12 | 55 |

**Table 4. Random feature exclusion analysis for COVID-19 classification: detailed groups of four-gene subsets**

| Group_Index | Selected Features to Drop |
| --- | --- |
| 1 | GPR15, PTGDR2, FCER1A, MAL |
| 2 | KANK2, S100B, SPEG, VNN1 |
| 3 | LIF, MAB21L2, S100B, TMOD1 |
| 4 | AQP1, HBB, LIF, MXI1 |
| 5 | AQP1, MAB21L2, MXI1, TNS1 |
| 6 | NAP1L3, SGCA, SNCA, SPINK2 |
| 7 | BAMBI, DNASE1L3, FCER1A, PTGDR2 |
| 8 | GPR15, MAB21L2, SGCA, VNN1 |
| 9 | GPR15, OLFM1, PTGDR2, SPEG |
| 10 | CYP4F3, FCER1A, LMOD1, TMOD1 |
| 11 | AKAP12, DNASE1L3, GPR15, SPEG |
| 12 | APOA2, FCER1A, IFITM1, LIF |
| 13 | HBB, PTGDR2, SPEG, VNN1 |
| 14 | NAP1L3, S100B, SNCA, TMOD1 |
| 15 | APOA2, GPR15, LIF, MAB21L2 |
| 16 | CA1, CYP4F3, HBB, NAP1L3 |
| 17 | FCER1A, LIF, S100B, TMOD1 |
| 18 | CYP4F3, DNASE1L3, MXI1, SPINK2 |
| 19 | APOA2, CYP4F3, PTGDR2, S100B |
| 20 | HBB, MAB21L2, PTGDR2, SNCA |
| 21 | CYP4F3, LIF, SGCA, TNS1 |
| 22 | COL9A3, FCER1A, TMOD1, TNS1 |
| 23 | APOA2, MAL, SGCA, VNN1 |
| 24 | AKAP12, MAB21L2, SGCA, TNS1 |
| 25 | AQP1, IFITM1, LIF, SPINK2 |
| 26 | DNASE1L3, GPR15, KANK2, VNN1 |
| 27 | IFITM1, LMOD1, P2RY14, SPINK2 |
| 28 | APOA2, BAMBI, HBB, IFITM1 |
| 29 | BAMBI, COL9A3, L1CAM, TMOD1 |
| 30 | LIF, MAL, MXI1, PTGDR2 |

| Group_Index | Selected Features to Drop |
| --- | --- |
| 1 | GPR15, PTGDR2, FCER1A, MAL |
| 2 | FCER1A, HBB, KANK2, OLFM1 |
| 3 | AQP1, BAMBI, CYP4F3, NAP1L3 |
| 4 | IFITM1, MAB21L2, MAL, SPINK2 |
| 5 | FCER1A, HBB, L1CAM, SPINK2 |
| 6 | CYP4F3, GPR15, HBB, S100B |
| 7 | APOA2, AQP1, MAB21L2, NAP1L3 |
| 8 | LMOD1, MAB21L2, MXI1, SNCA |
| 9 | BAMBI, CA1, LMOD1, PTGDR2 |
| 10 | IFITM1, L1CAM, MAB21L2, TNS1 |
| 11 | APOA2, MAB21L2, SPEG, SPINK2 |
| 12 | APOA2, IFITM1, P2RY14, TNS1 |
| 13 | APOA2, CYP4F3, S100B, SPEG |
| 14 | APOA2, CYP4F3, DNASE1L3, OLFM1 |
| 15 | AQP1, BAMBI, MAL, NAP1L3 |
| 16 | AKAP12, AQP1, GPR15, TMOD1 |
| 17 | AQP1, BAMBI, GPR15, MAL |
| 18 | IFITM1, KANK2, L1CAM, OLFM1 |
| 19 | AQP1, P2RY14, SNCA, SPINK2 |
| 20 | APOA2, LIF, NAP1L3, VNN1 |
| 21 | MAB21L2, OLFM1, S100B, TNS1 |
| 22 | GPR15, PTGDR2, SPINK2, TMOD1 |
| 23 | AKAP12, APOA2, L1CAM, PTGDR2 |
| 24 | APOA2, BAMBI, CYP4F3, P2RY14 |
| 25 | OLFM1, P2RY14, SPEG, VNN1 |
| 26 | MAL, NAP1L3, P2RY14, SPEG |
| 27 | CA1, HBB, LMOD1, NAP1L3 |
| 28 | KANK2, S100B, SNCA, VNN1 |
| 29 | MAL, OLFM1, S100B, VNN1 |
| 30 | AQP1, P2RY14, TMOD1, TNS1 |

**Table 5. Random feature exclusion analysis for CRC classification: detailed groups of four-gene subsets**

**
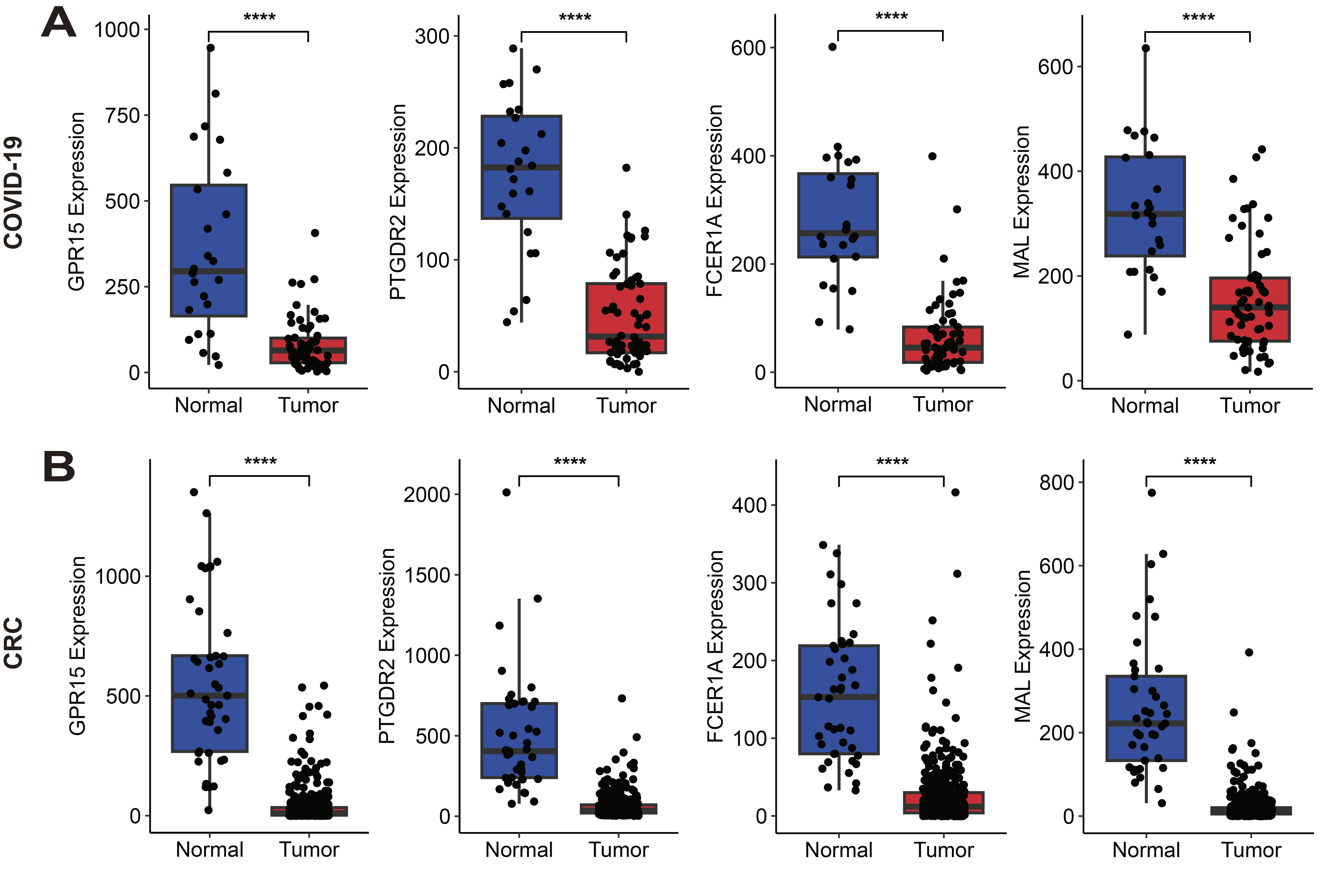
**

Figure 1. Box plots of potential core hub gene expression in two datasets.

**A) Differential expression of potential core hub genes in the COVID-19 dataset. B) Differential expression of potential core hub genes in the CRC dataset.**

**
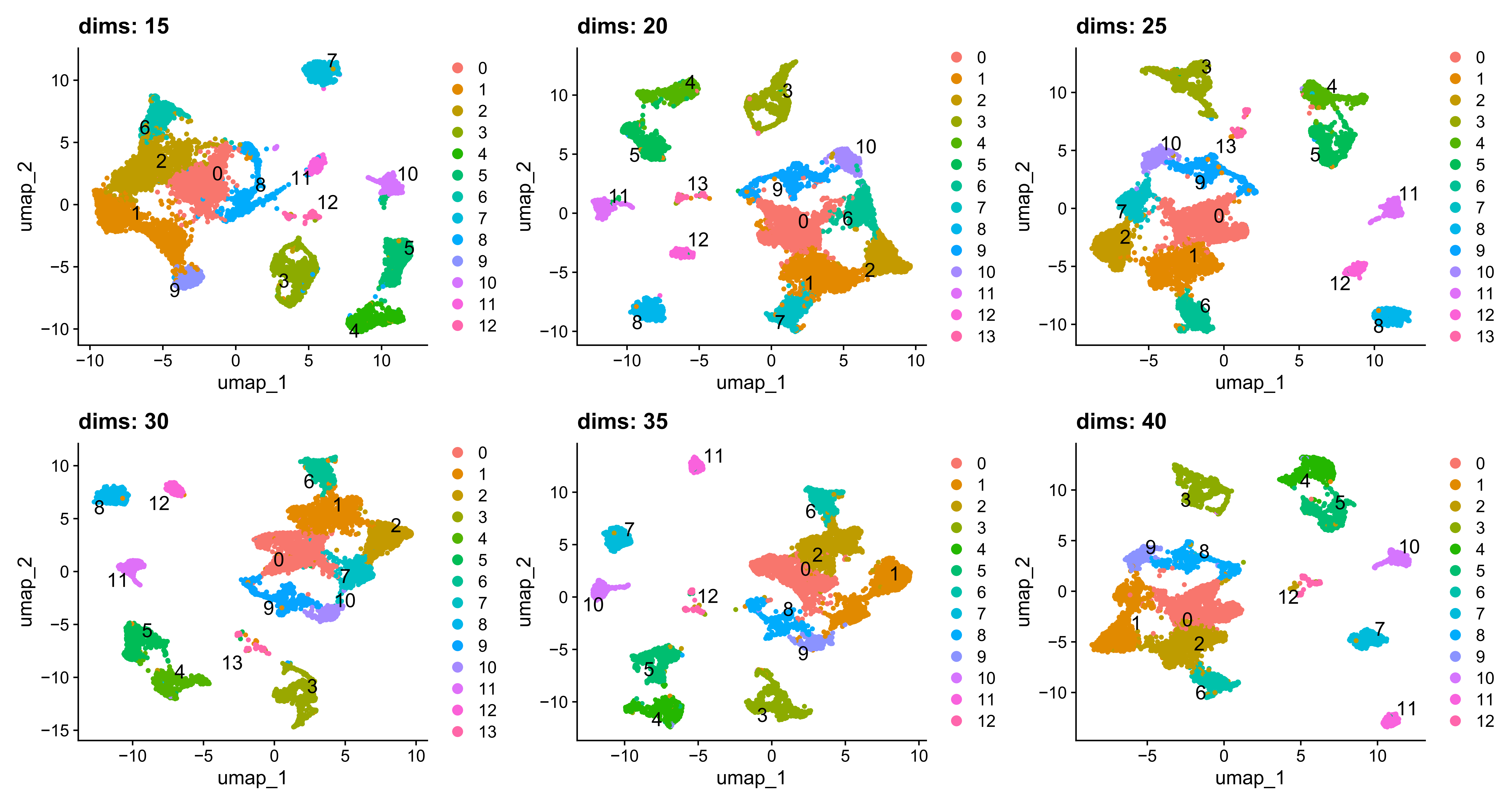
**

Figure 2. The UMAP clustering results under different dims parameters, with dims set to 15, 20, 25, 30, 35, and 40.


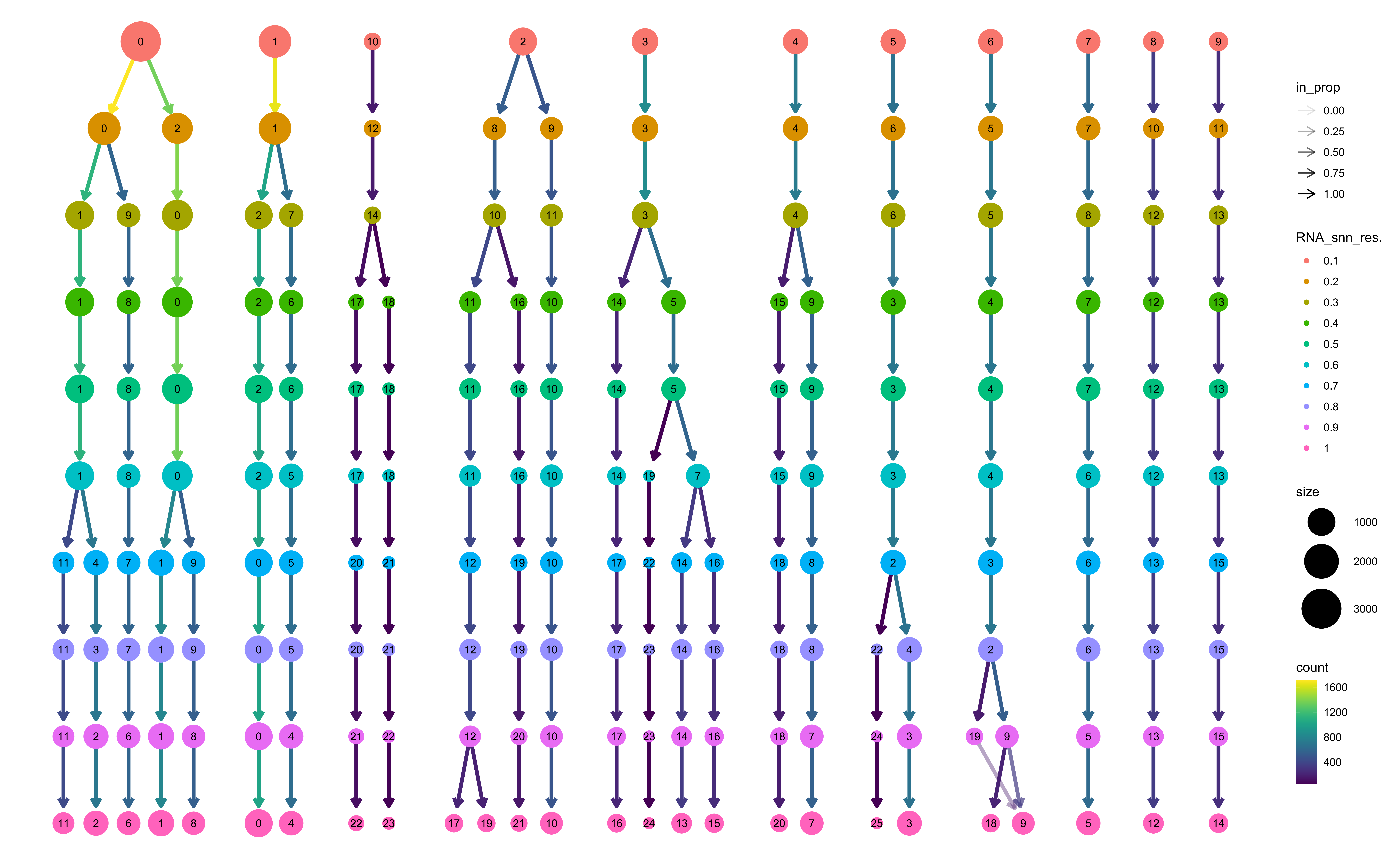


Figure 3. The cell clustering and lineage trajectory maps under different resolution parameters, with different colors representing different resolutions.

**
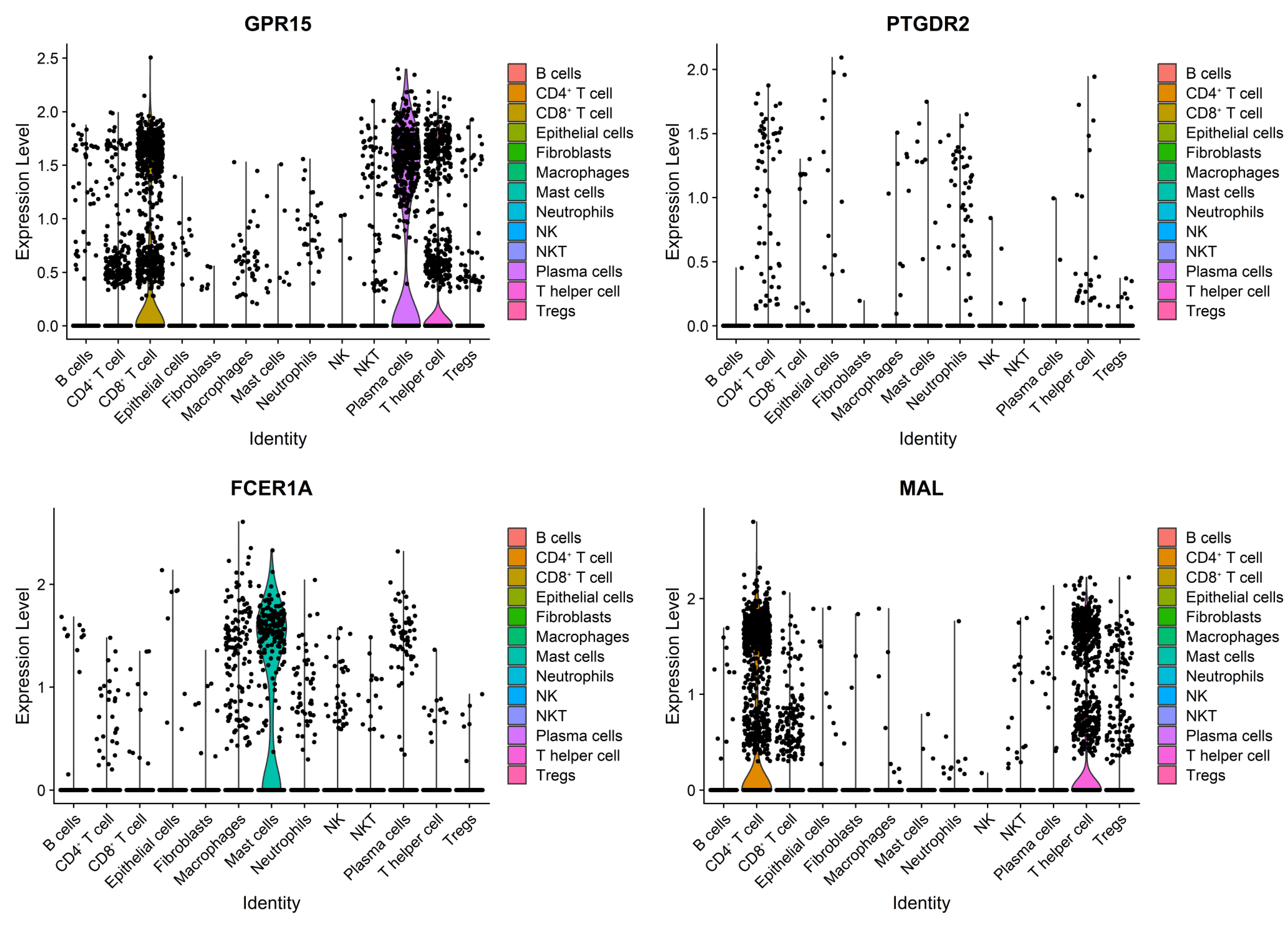
**

Figure 4. The violin plots showing the distribution of different genes across various cell types.

Table 6. Scores of MicroRNAs (miRNAs)

| miRNA | Score |
| --- | --- |
| miR-3619-5p | 3.85 |
| miR-761 | 1.85 |
| miR-449c-5p | 1.85 |
| miR-125b-5p | 1.67 |
| miR-507 | 1 |
| miR-22-3p | 1 |
| miR-193a-3p | 0.94 |
| miR-4319 | 0.92 |
| miR-338-3p | 0.92 |
| miR-125a-5p | 0.92 |
| miR-107 | 0.92 |
| miR-27a-3p | 0.85 |
| miR-24-3p | 0.85 |
| miR-129-5p | 0.85 |

Table 7. Scores of transcription factors (TFs)

| TF | S_c_ | Symbol | a | S_WODC_ | Score |
| --- | --- | --- | --- | --- | --- |
| p53 | 4 | 1 | 1 | 7.19 | 7.19 |
| MYB | 3 | 1 | 0.5 | 5.17 | 2.58 |
| MITF | 3 | -1 | 0.5 | 2.03 | -1.02 |
| ZIC2 | 2 | 1 | 0.2 | 3.52 | 0.70 |
| MEF2D | 1 | 1 | 0 | 6.00 | 0 |
| TFE3 | 1 | 1 | 0 | 4.99 | 0 |
| p63 | 1 | 1 | 0 | 2.93 | 0 |
| HIC1 | 1 | 1 | 0 | 2.92 | 0 |
| SOX4 | 1 | -1 | 0 | 2.84 | 0 |
| SOX2 | 1 | 1 | 0 | 2.76 | 0 |

Table 8. Binding Energies between small molecule drug and target protein

| Drug | GPR15 (kcal/mol) | PTGDR2 (kcal/mol) | FCER1A (kcal/mol) | MAL (kcal/mol) |
| --- | --- | --- | --- | --- |
| Medrysone | -7.08 | -8.92 | -5.49 | -6.53 |
| E-4031 | -5.84 | -7.67 | -6.42 | -6.54 |
| Berberine | -5.98 | -7.64 | -4.63 | -5.74 |
| Rimexolone | -6.67 | -8.89 | -5.93 | -6.95 |
| MG-132 | -4.23 | -6.46 | -3.42 | -2.69 |
| LDN-57444 | -6.66 | -8.69 | -4.42 | -2.65 |


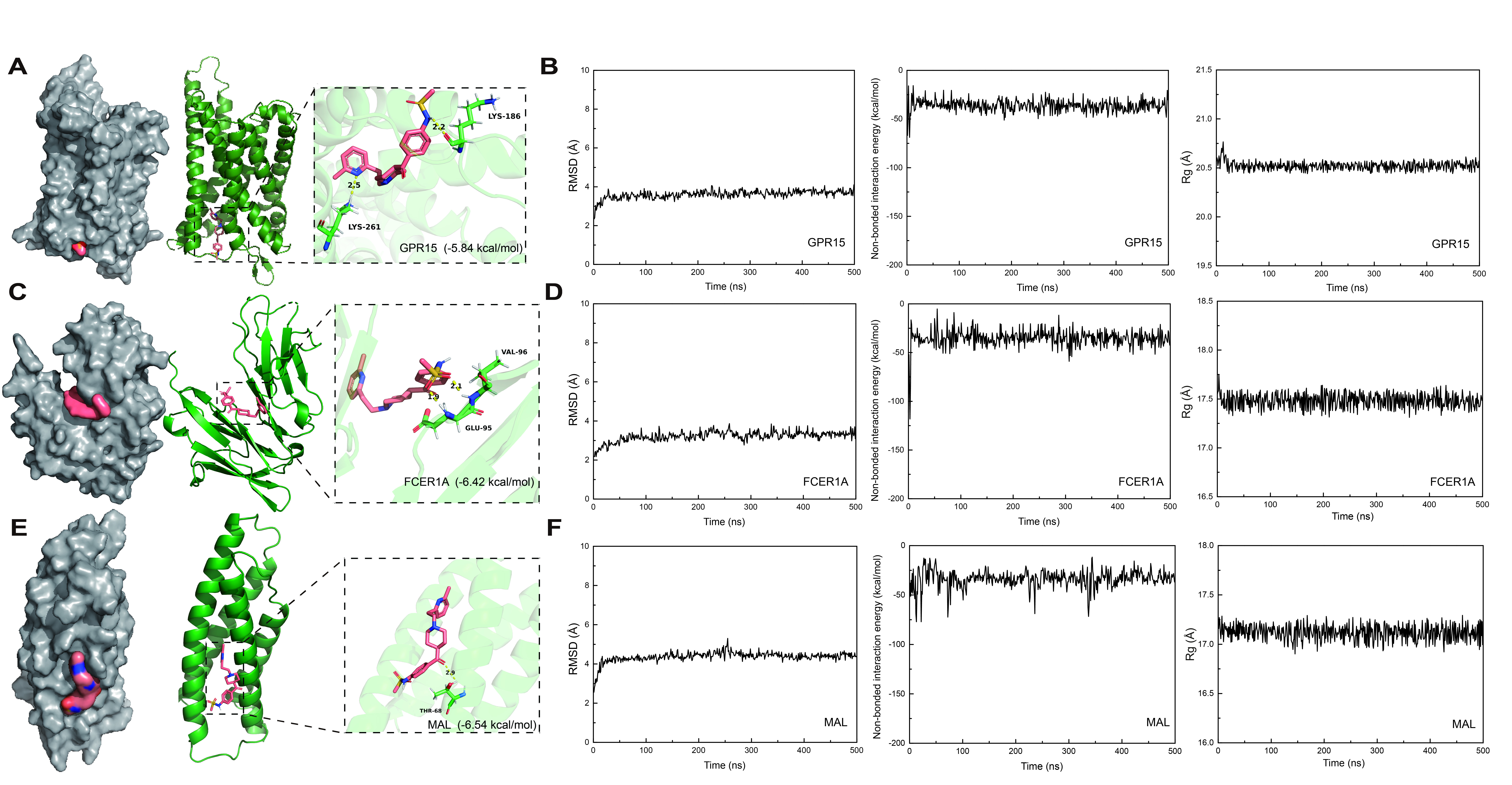


Figure 5. GPR15, FCER1A, and MAL docking scores and molecular dynamics simulations.

A) Docking results of E-4031 with GPR15. B) Molecular dynamics simulation results for E-4031 with GPR15. C) Docking results of E-4031 with FCER1A. D) Molecular dynamics simulation results for E-4031 with FCER1A. E) Docking results of E-4031 with MAL. F) Molecular dynamics simulation results for E-4031 with MAL.

**References**

1. M.E. Ritchie, B. Phipson, D. Wu, Y. Hu, C.W. Law, W. Shi, G.K. Smyth, limma powers differential expression analyses for RNA-sequencing and microarray studies, Nucleic Acids Research 43 (2015) e47–e47.
2. T. Wu, E. Hu, S. Xu, M. Chen, P. Guo, Z. Dai, T. Feng, L. Zhou, W. Tang, L. Zhan, X. Fu, S. Liu, X. Bo, G. Yu, clusterProfiler 4.0: A universal enrichment tool for interpreting omics data, The Innovation 2 (2021).
3. L. Zhang, Z. Li, K.M. Skrzypczynska, Q. Fang, W. Zhang, S.A. O’Brien, Y. He, L. Wang, Q. Zhang, A. Kim, others, Single-cell analyses inform mechanisms of myeloid-targeted therapies in colon cancer, Cell 181 (2020) 442–459.
4. T. Stuart, A. Butler, P. Hoffman, C. Hafemeister, E. Papalexi, W.M. Mauck, Y. Hao, M. Stoeckius, P. Smibert, R. Satija, Comprehensive integration of single-cell data, Cell 177 (2019) 1888–1902.
5. A.M. Newman, C.L. Liu, M.R. Green, A.J. Gentles, W. Feng, Y. Xu, C.D. Hoang, M. Diehn, A.A. Alizadeh, Robust enumeration of cell subsets from tissue expression profiles, Nature Methods 12 (2015) 453–457.
6. M. Hill, N. Tran, miRNA interplay: mechanisms and consequences in cancer, Disease Models &Amp; Mechanisms 14 (2021).
7. J.M. Vaquerizas, S.K. Kummerfeld, S.A. Teichmann, N.M. Luscombe, A census of human transcription factors: Function, expression and evolution, Nature Reviews Genetics 22 (2021) 21–36.
8. A. Jeggari, D.S. Marks, E. Larsson, miRcode: a map of putative microRNA target sites in the long non-coding transcriptome, Bioinformatics 28 (2012) 2062–2063.
9. H. Dweep, C. Sticht, P. Pandey, N. Gretz, miRWalk–database: Prediction of possible miRNA binding sites by "walking" the genes of three genomes, Journal of Biomedical Informatics 44 (2011) 839–847.
10. C. Feng, C. Song, S. Song, G. Zhang, M. Yin, Y. Zhang, F. Qian, Q. Wang, M. Guo, C. Li, KnockTF 2.0: A comprehensive gene expression profile database with knockdown/knockout of transcription (co-) factors in multiple species, Nucleic Acids Research 52 (2024) D183–D193.
11. D. Szklarczyk, R. Kirsch, M. Koutrouli, K. Nastou, F. Mehryary, R. Hachilif, A.L. Gable, T. Fang, Nadezhda.T. Doncheva, S. Pyysalo, P. Bork, Lars.J. Jensen, C. von.Mering, The STRING database in 2023: protein–protein association networks and functional enrichment analyses for any sequenced genome of interest, Nucleic Acids Research 51 (2022) D638–D646.
